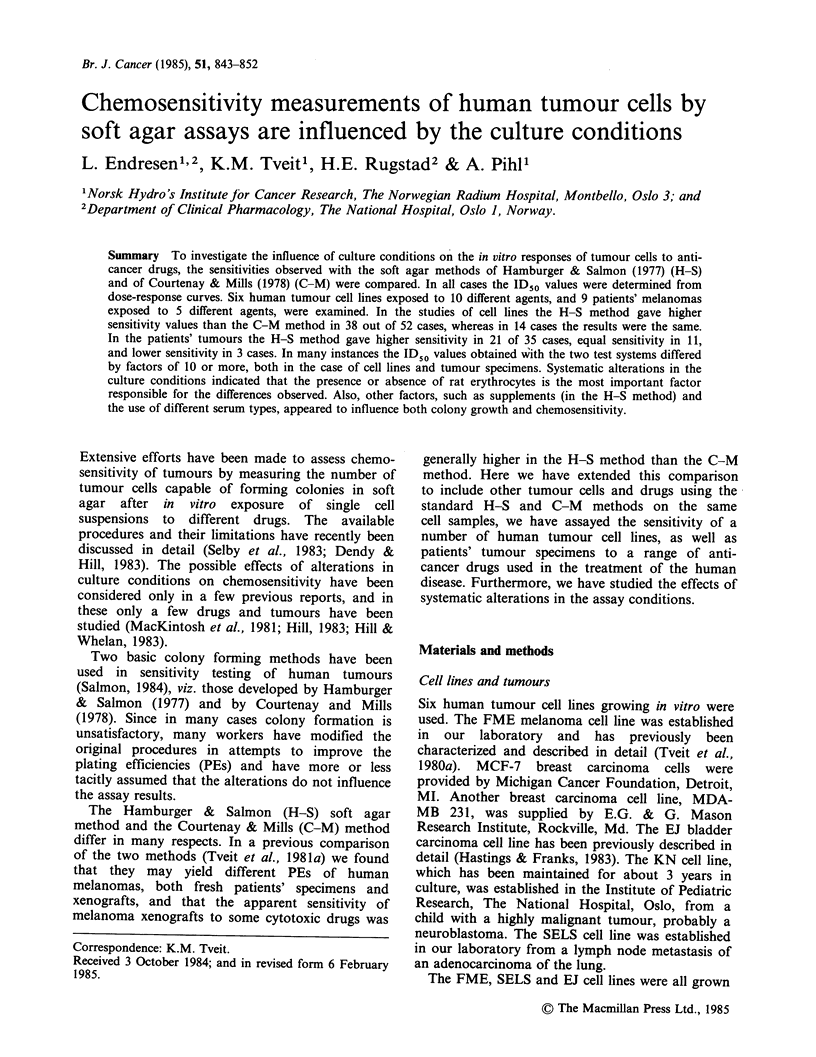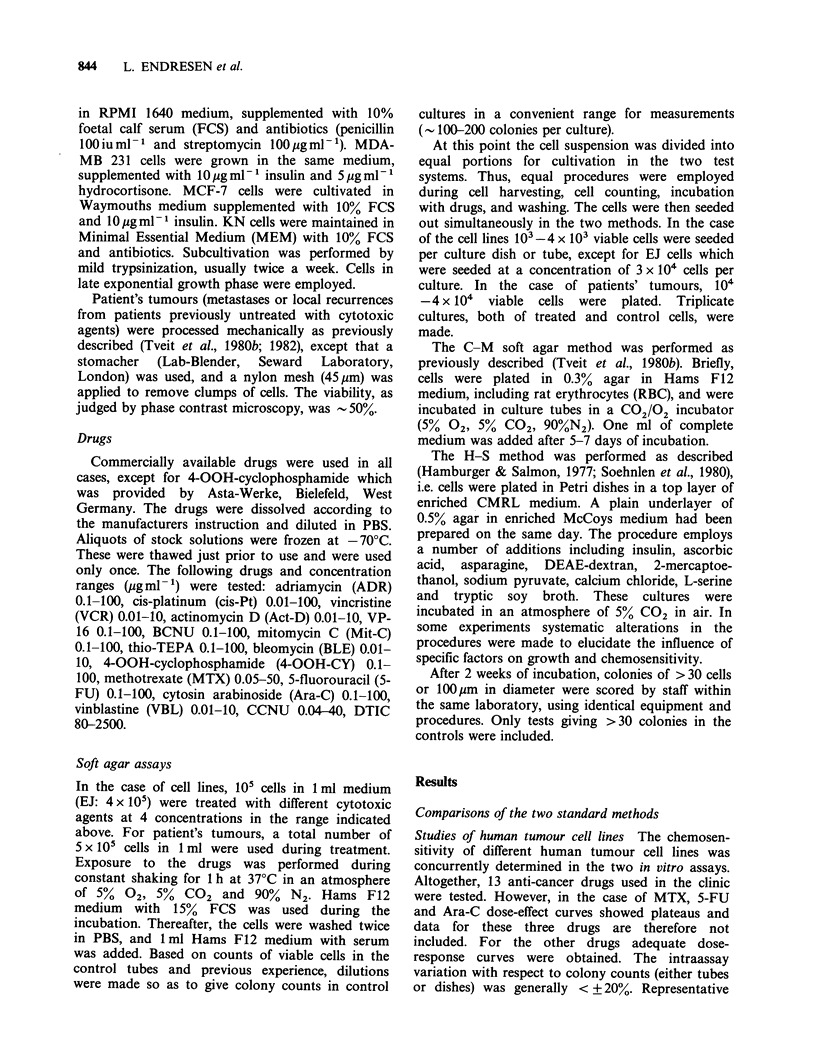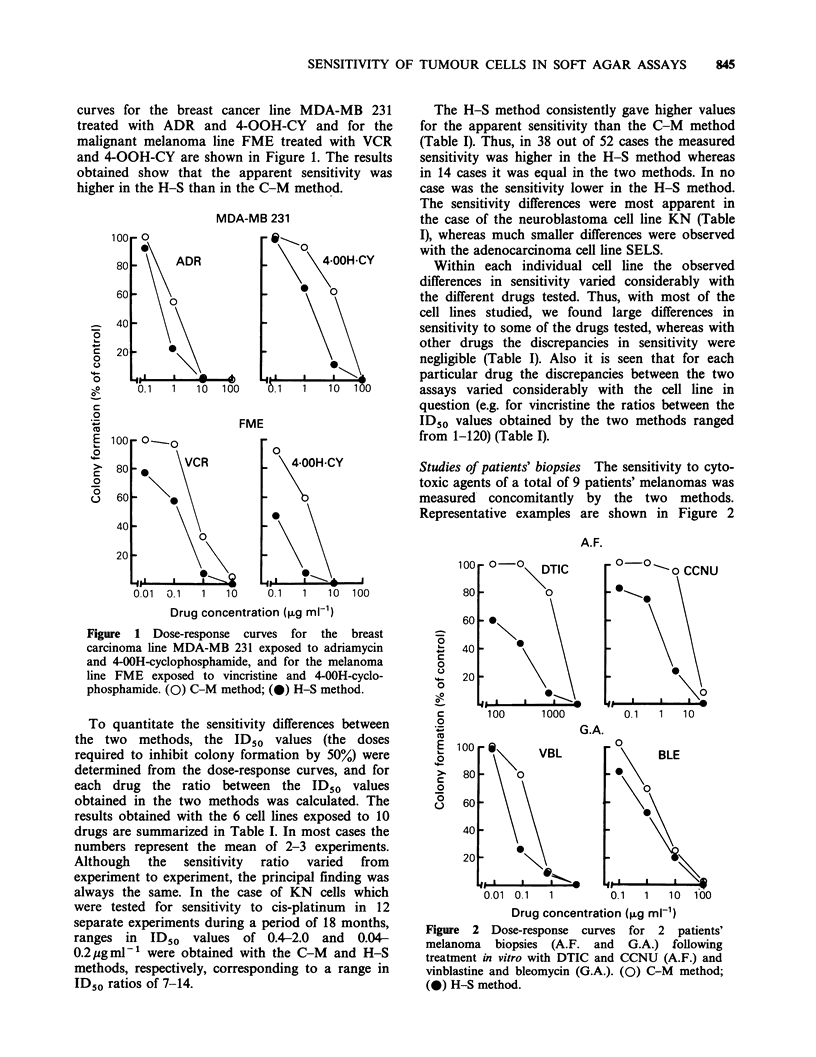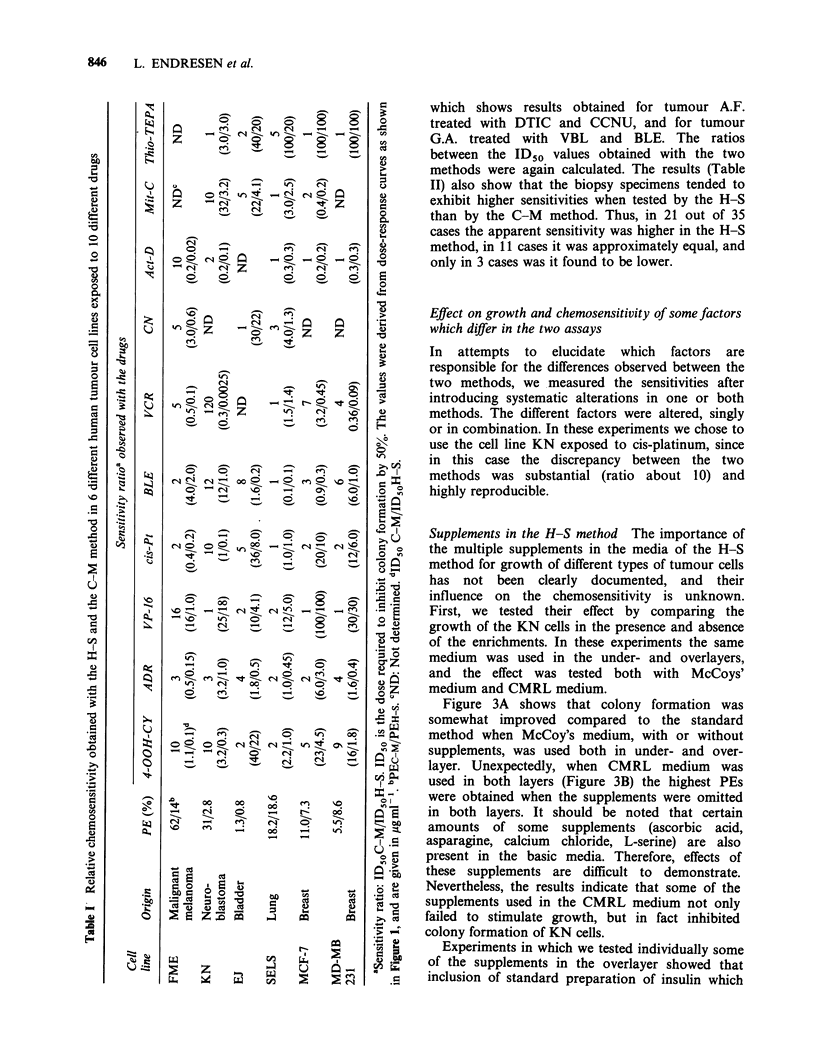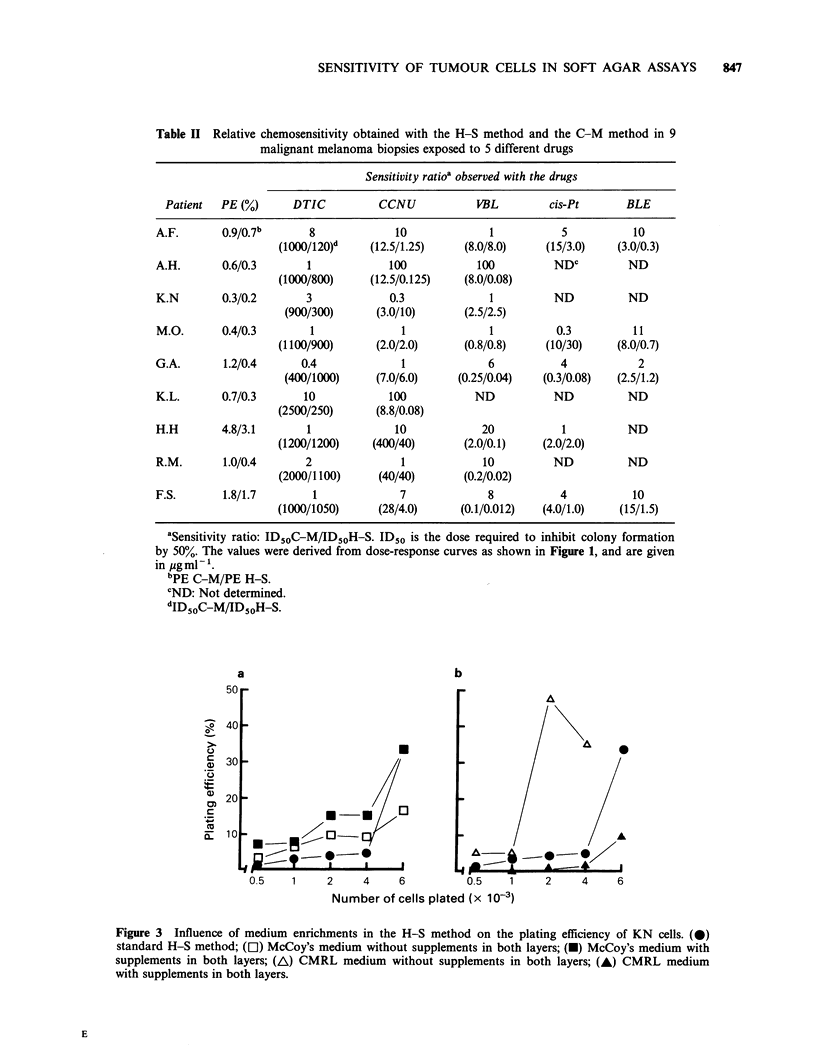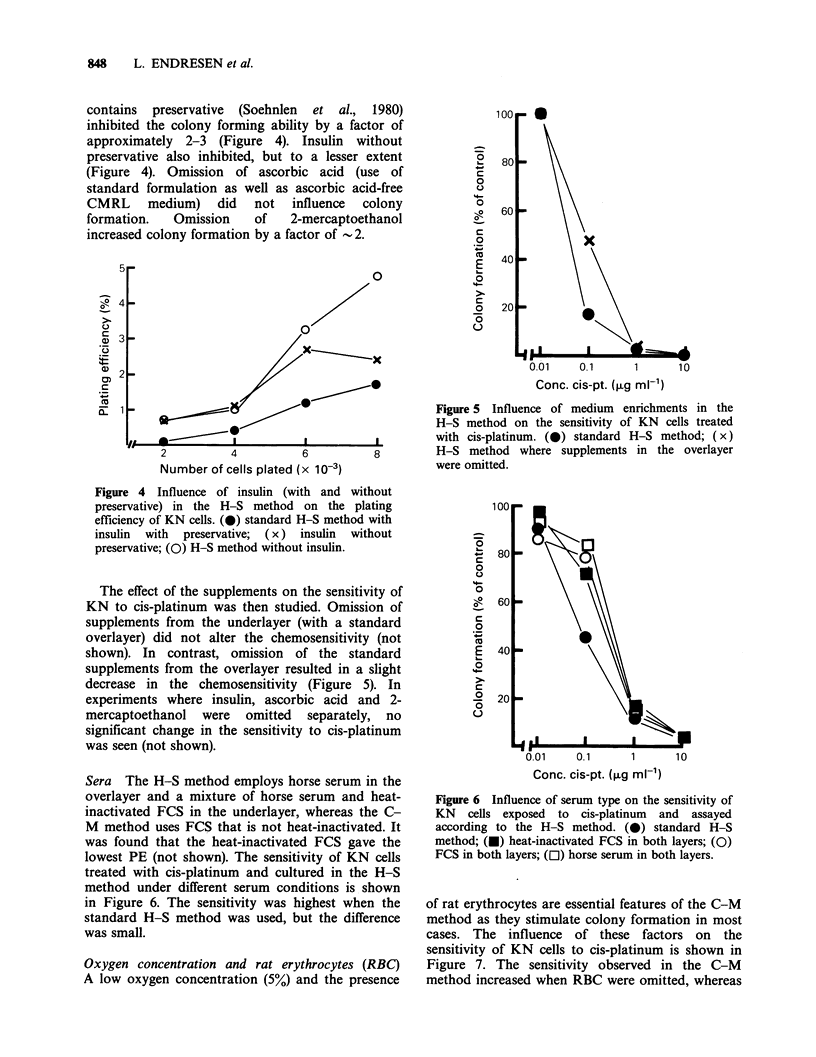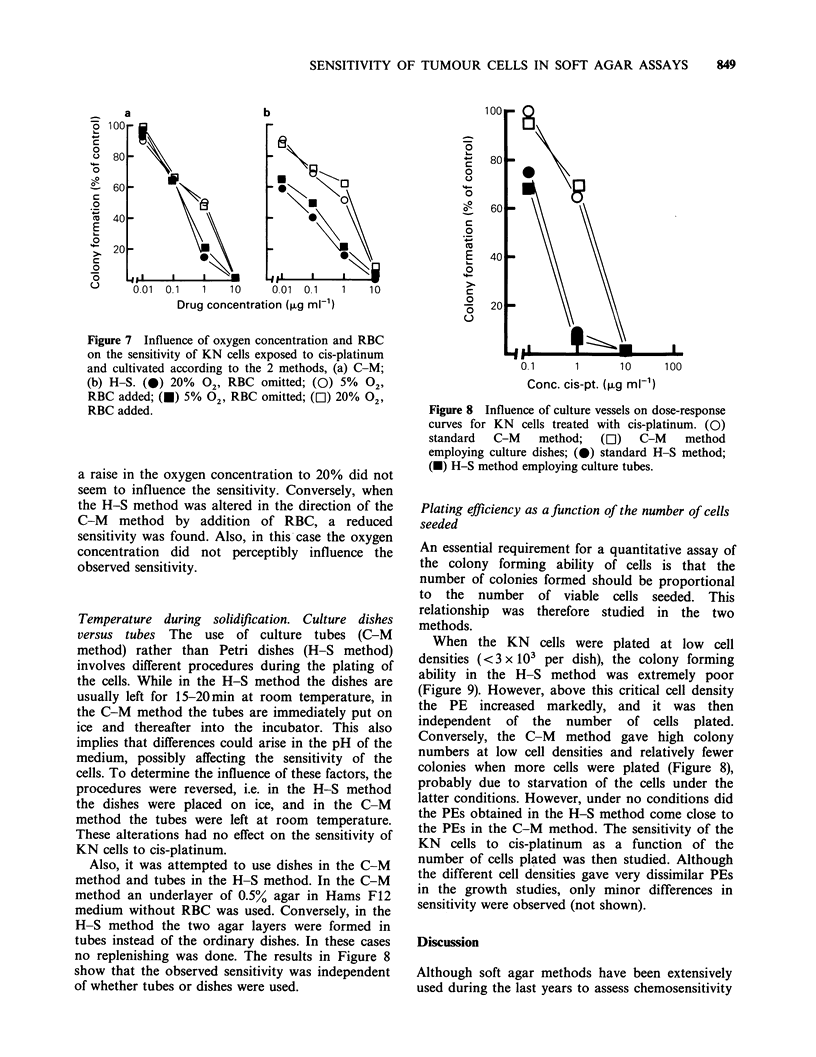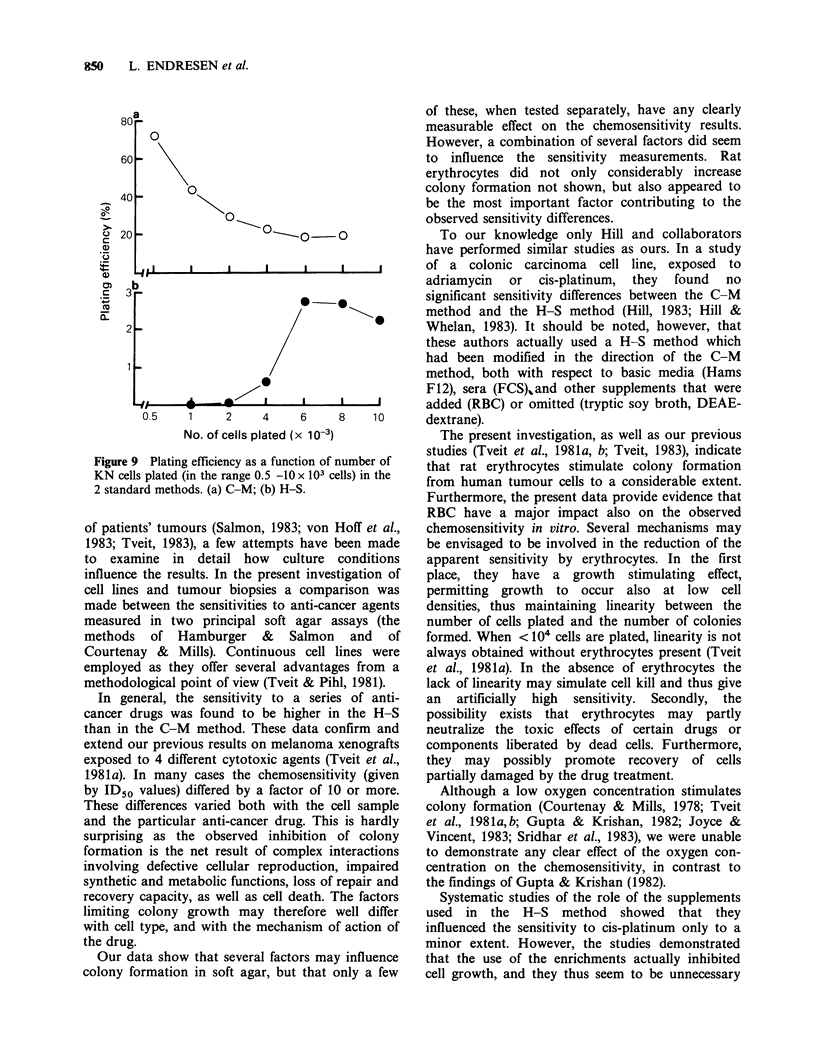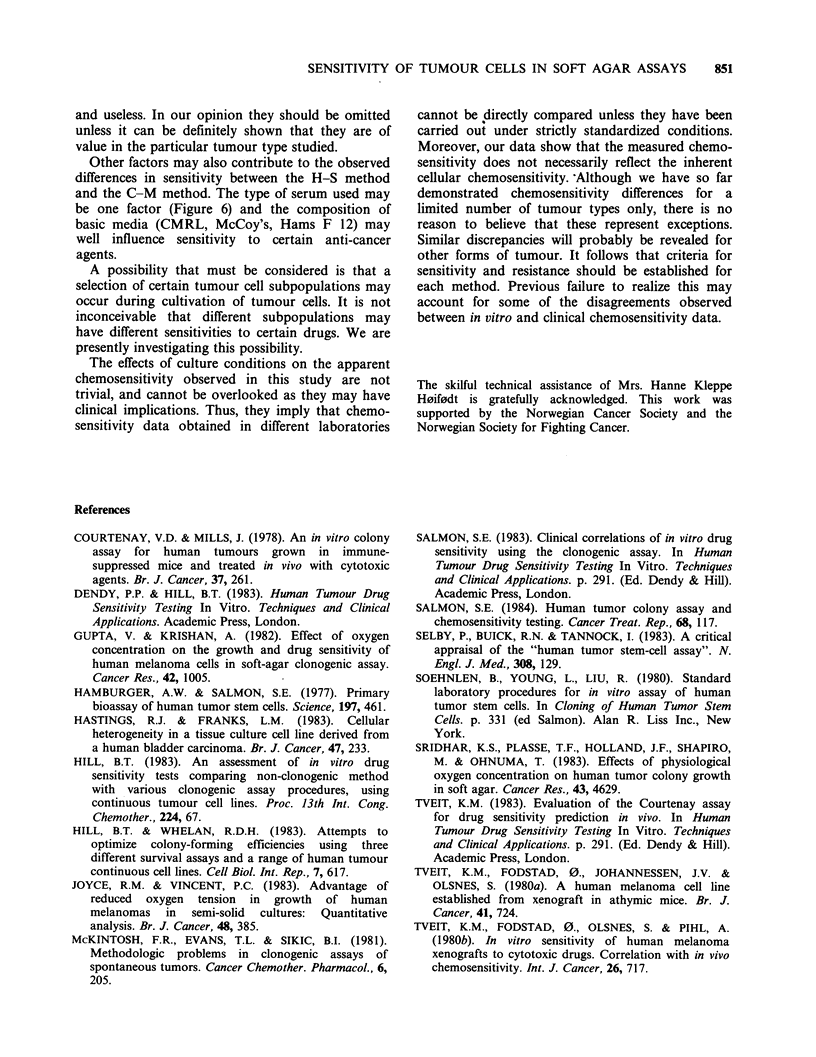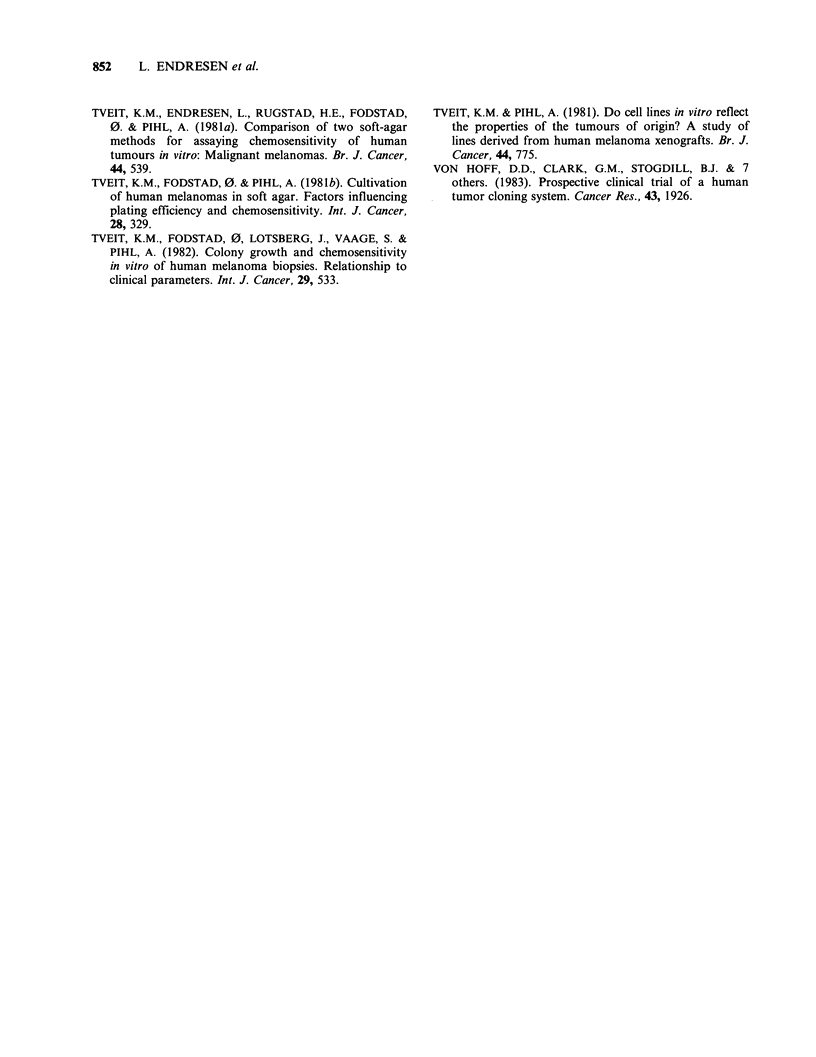# Chemosensitivity measurements of human tumour cells by soft agar assays are influenced by the culture conditions.

**DOI:** 10.1038/bjc.1985.130

**Published:** 1985-06

**Authors:** L. Endresen, K. M. Tveit, H. E. Rugstad, A. Pihl

## Abstract

To investigate the influence of culture conditions on the in vitro responses of tumour cells to anticancer drugs, the sensitivities observed with the soft agar methods of Hamburger & Salmon (1977) (H-S) and of Courtenay & Mills (1978) (C-M) were compared. In all cases the ID50 values were determined from dose-response curves. Six human tumour cell lines exposed to 10 different agents, and 9 patients' melanomas exposed to 5 different agents, were examined. In the studies of cell lines the H-S method gave higher sensitivity values than the C-M method in 38 out of 52 cases, whereas in 14 cases the results were the same. In the patients' tumours the H-S method gave higher sensitivity in 21 of 35 cases, equal sensitivity in 11, and lower sensitivity in 3 cases. In many instances the ID50 values obtained with the two test systems differed by factors of 10 or more, both in the case of cell lines and tumour specimens. Systematic alterations in the culture conditions indicated that the presence or absence of rat erythrocytes is the most important factor responsible for the differences observed. Also, other factors, such as supplements (in the H-S method) and the use of different serum types, appeared to influence both colony growth and chemosensitivity.


*Br. J. Cancer* (1985), **51**, 843–852

### Chemosensitivity measurements of human tumour cells by soft agar assays are influenced by the culture conditions

L. Endresen[1,2], K.M. Tveit[1], H.E. Rugstad[2] & A. Pihl[1]

[1]*Norsk Hydro's Institute for Cancer Research, The Norwegian Radium Hospital, Montbello, Oslo 3; and* [2]*Department of Clinical Pharmacology, The National Hospital, Oslo 1, Norway.*

**Summary** To investigate the influence of culture conditions on the *in vitro* responses of tumour cells to anti-cancer drugs, the sensitivities observed with the soft agar methods of Hamburger & Salmon (1977) (H–S) and of Courtenay & Mills (1978) (C–M) were compared. In all cases the $ID_{50}$ values were determined from dose-response curves. Six human tumour cell lines exposed to 10 different agents, and 9 patients' melanomas exposed to 5 different agents, were examined. In the studies of cell lines the H–S method gave higher sensitivity values than the C–M method in 38 out of 52 cases, whereas in 14 cases the results were the same. In the patients' tumours the H–S method gave higher sensitivity in 21 of 35 cases, equal sensitivity in 11, and lower sensitivity in 3 cases. In many instances the $ID_{50}$ values obtained with the two test systems differed by factors of 10 or more, both in the case of cell lines and tumour specimens. Systematic alterations in the culture conditions indicated that the presence or absence of rat erythrocytes is the most important factor responsible for the differences observed. Also, other factors, such as supplements (in the H–S method) and the use of different serum types, appeared to influence both colony growth and chemosensitivity.

Extensive efforts have been made to assess chemosensitivity of tumours by measuring the number of tumour cells capable of forming colonies in soft agar after *in vitro* exposure of single cell suspensions to different drugs. The available procedures and their limitations have recently been discussed in detail (Selby *et al.*, 1983; Dendy & Hill, 1983). The possible effects of alterations in culture conditions on chemosensitivity have been considered only in a few previous reports, and in these only a few drugs and tumours have been studied (MacKintosh *et al.*, 1981; Hill, 1983; Hill & Whelan, 1983).

Two basic colony forming methods have been used in sensitivity testing of human tumours (Salmon, 1984), *viz.* those developed by Hamburger & Salmon (1977) and by Courtenay and Mills (1978). Since in many cases colony formation is unsatisfactory, many workers have modified the original procedures in attempts to improve the plating efficiencies (PEs) and have more or less tacitly assumed that the alterations do not influence the assay results.

The Hamburger & Salmon (H–S) soft agar method and the Courtenay & Mills (C–M) method differ in many respects. In a previous comparison of the two methods (Tveit *et al.*, 1981*a*) we found that they may yield different PEs of human melanomas, both fresh patients' specimens and xenografts, and that the apparent sensitivity of melanoma xenografts to some cytotoxic drugs was generally higher in the H–S method than the C–M method. Here we have extended this comparison to include other tumour cells and drugs using the standard H–S and C–M methods on the same cell samples, we have assayed the sensitivity of a number of human tumour cell lines, as well as patients' tumour specimens to a range of anti-cancer drugs used in the treatment of the human disease. Furthermore, we have studied the effects of systematic alterations in the assay conditions.

Correspondence: K.M. Tveit.
Received 3 October 1984; and in revised form 6 February 1985.

### Materials and methods

### *Cell lines and tumours*

Six human tumour cell lines growing *in vitro* were used. The FME melanoma cell line was established in our laboratory and has previously been characterized and described in detail (Tveit *et al.*, 1980*a*). MCF-7 breast carcinoma cells were provided by Michigan Cancer Foundation, Detroit, MI. Another breast carcinoma cell line, MDA-MB 231, was supplied by E.G. & G. Mason Research Institute, Rockville, Md. The EJ bladder carcinoma cell line has been previously described in detail (Hastings & Franks, 1983). The KN cell line, which has been maintained for about 3 years in culture, was established in the Institute of Pediatric Research, The National Hospital, Oslo, from a child with a highly malignant tumour, probably a neuroblastoma. The SELS cell line was established in our laboratory from a lymph node metastasis of an adenocarcinoma of the lung.

The FME, SELS and EJ cell lines were all grown

© The Macmillan Press Ltd., 1985

in RPMI 1640 medium, supplemented with 10% foetal calf serum (FCS) and antibiotics (penicillin $100\,iu\,ml^{-1}$ and streptomycin $100\,\mu g\,ml^{-1}$). MDA-MB 231 cells were grown in the same medium, supplemented with $10\,\mu g\,ml^{-1}$ insulin and $5\,\mu g\,ml^{-1}$ hydrocortisone. MCF-7 cells were cultivated in Waymouths medium supplemented with 10% FCS and $10\,\mu g\,ml^{-1}$ insulin. KN cells were maintained in Minimal Essential Medium (MEM) with 10% FCS and antibiotics. Subcultivation was performed by mild trypsinization, usually twice a week. Cells in late exponential growth phase were employed.

Patient's tumours (metastases or local recurrences from patients previously untreated with cytotoxic agents) were processed mechanically as previously described (Tveit *et al.*, 1980*b*; 1982), except that a stomacher (Lab-Blender, Seward Laboratory, London) was used, and a nylon mesh ($45\,\mu m$) was applied to remove clumps of cells. The viability, as judged by phase contrast microscopy, was ~50%.

*Drugs*

Commercially available drugs were used in all cases, except for 4-OOH-cyclophosphamide which was provided by Asta-Werke, Bielefeld, West Germany. The drugs were dissolved according to the manufacturers instruction and diluted in PBS. Aliquots of stock solutions were frozen at −70°C. These were thawed just prior to use and were used only once. The following drugs and concentration ranges ($\mu g\,ml^{-1}$) were tested: adriamycin (ADR) 0.1–100, cis-platinum (cis-Pt) 0.01–100, vincristine (VCR) 0.01–10, actinomycin D (Act-D) 0.01–10, VP-16 0.1–100, BCNU 0.1–100, mitomycin C (Mit-C) 0.1–100, thio-TEPA 0.1–100, bleomycin (BLE) 0.01–10, 4-OOH-cyclophosphamide (4-OOH-CY) 0.1–100, methotrexate (MTX) 0.05–50, 5-fluorouracil (5-FU) 0.1–100, cytosin arabinoside (Ara-C) 0.1–100, vinblastine (VBL) 0.01–10, CCNU 0.04–40, DTIC 80–2500.

*Soft agar assays*

In the case of cell lines, $10^5$ cells in 1 ml medium (EJ: $4\times10^5$) were treated with different cytotoxic agents at 4 concentrations in the range indicated above. For patient's tumours, a total number of $5\times10^5$ cells in 1 ml were used during treatment. Exposure to the drugs was performed during constant shaking for 1 h at 37°C in an atmosphere of 5% $O_2$, 5% $CO_2$ and 90% $N_2$. Hams F12 medium with 15% FCS was used during the incubation. Thereafter, the cells were washed twice in PBS, and 1 ml Hams F12 medium with serum was added. Based on counts of viable cells in the control tubes and previous experience, dilutions were made so as to give colony counts in control cultures in a convenient range for measurements (~100–200 colonies per culture).

At this point the cell suspension was divided into equal portions for cultivation in the two test systems. Thus, equal procedures were employed during cell harvesting, cell counting, incubation with drugs, and washing. The cells were then seeded out simultaneously in the two methods. In the case of the cell lines $10^3-4\times10^3$ viable cells were seeded per culture dish or tube, except for EJ cells which were seeded at a concentration of $3\times10^4$ cells per culture. In the case of patients' tumours, $10^4-4\times10^4$ viable cells were plated. Triplicate cultures, both of treated and control cells, were made.

The C–M soft agar method was performed as previously described (Tveit *et al.*, 1980*b*). Briefly, cells were plated in 0.3% agar in Hams F12 medium, including rat erythrocytes (RBC), and were incubated in culture tubes in a $CO_2/O_2$ incubator (5% $O_2$, 5% $CO_2$, 90%$N_2$). One ml of complete medium was added after 5–7 days of incubation.

The H–S method was performed as described (Hamburger & Salmon, 1977; Soehnlen *et al.*, 1980), i.e. cells were plated in Petri dishes in a top layer of enriched CMRL medium. A plain underlayer of 0.5% agar in enriched McCoys medium had been prepared on the same day. The procedure employs a number of additions including insulin, ascorbic acid, asparagine, DEAE-dextran, 2-mercaptoethanol, sodium pyruvate, calcium chloride, L-serine and tryptic soy broth. These cultures were incubated in an atmosphere of 5% $CO_2$ in air. In some experiments systematic alterations in the procedures were made to elucidate the influence of specific factors on growth and chemosensitivity.

After 2 weeks of incubation, colonies of >30 cells or $100\,\mu m$ in diameter were scored by staff within the same laboratory, using identical equipment and procedures. Only tests giving >30 colonies in the controls were included.

### Results

*Comparisons of the two standard methods*

*Studies of human tumour cell lines* The chemosensitivity of different human tumour cell lines was concurrently determined in the two *in vitro* assays. Altogether, 13 anti-cancer drugs used in the clinic were tested. However, in the case of MTX, 5-FU and Ara-C dose-effect curves showed plateaus and data for these three drugs are therefore not included. For the other drugs adequate dose-response curves were obtained. The intraassay variation with respect to colony counts (either tubes or dishes) was generally $<\pm20\%$. Representative curves for the breast cancer line MDA-MB 231 treated with ADR and 4-OOH-CY and for the malignant melanoma line FME treated with VCR and 4-OOH-CY are shown in Figure 1. The results obtained show that the apparent sensitivity was higher in the H–S than in the C–M method.

**Figure 1** Dose-response curves for the breast carcinoma line MDA-MB 231 exposed to adriamycin and 4-00H-cyclophosphamide, and for the melanoma line FME exposed to vincristine and 4-00H-cyclophosphamide. (○) C–M method; (●) H–S method.

To quantitate the sensitivity differences between the two methods, the $ID_{50}$ values (the doses required to inhibit colony formation by 50%) were determined from the dose-response curves, and for each drug the ratio between the $ID_{50}$ values obtained in the two methods was calculated. The results obtained with the 6 cell lines exposed to 10 drugs are summarized in Table I. In most cases the numbers represent the mean of 2–3 experiments. Although the sensitivity ratio varied from experiment to experiment, the principal finding was always the same. In the case of KN cells which were tested for sensitivity to cis-platinum in 12 separate experiments during a period of 18 months, ranges in $ID_{50}$ values of 0.4–2.0 and 0.04–0.2 $\mu g\, ml^{-1}$ were obtained with the C–M and H–S methods, respectively, corresponding to a range in $ID_{50}$ ratios of 7–14.

The H–S method consistently gave higher values for the apparent sensitivity than the C–M method (Table I). Thus, in 38 out of 52 cases the measured sensitivity was higher in the H–S method whereas in 14 cases it was equal in the two methods. In no case was the sensitivity lower in the H–S method. The sensitivity differences were most apparent in the case of the neuroblastoma cell line KN (Table I), whereas much smaller differences were observed with the adenocarcinoma cell line SELS.

Within each individual cell line the observed differences in sensitivity varied considerably with the different drugs tested. Thus, with most of the cell lines studied, we found large differences in sensitivity to some of the drugs tested, whereas with other drugs the discrepancies in sensitivity were negligible (Table I). Also it is seen that for each particular drug the discrepancies between the two assays varied considerably with the cell line in question (e.g. for vincristine the ratios between the $ID_{50}$ values obtained by the two methods ranged from 1–120) (Table I).

*Studies of patients' biopsies* The sensitivity to cytotoxic agents of a total of 9 patients' melanomas was measured concomitantly by the two methods. Representative examples are shown in Figure 2

**Figure 2** Dose-response curves for 2 patients' melanoma biopsies (A.F. and G.A.) following treatment *in vitro* with DTIC and CCNU (A.F.) and vinblastine and bleomycin (G.A.). (○) C–M method; (●) H–S method.

**Table I** Relative chemosensitivity obtained with the H–S and the C–M method in 6 different human tumour cell lines exposed to 10 different drugs

| Cell line | Origin | PE (%) | Sensitivity ratio[a] observed with the drugs | | | | | | | | | |
|---|---|---|---|---|---|---|---|---|---|---|---|---|
| | | | 4-OOH-CY | ADR | VP-16 | cis-Pt | BLE | VCR | CN | Act-D | Mit-C | Thio-TEPA |
| FME | Malignant melanoma | 62/14[b] | 10 (1.1/0.1)[d] | 3 (0.5/0.15) | 16 (16/1.0) | 2 (0.4/0.2) | 2 (4.0/2.0) | 5 (0.5/0.1) | 5 (3.0/0.6) | 10 (0.2/0.02) | ND[c] | ND |
| KN | Neuro-blastoma | 31/2.8 | 10 (3.2/0.3) | 3 (3.2/1.0) | 1 (25/18) | 10 (1/0.1) | 12 (12/1.0) | 120 (0.3/0.0025) | ND | 2 (0.2/0.1) | 10 (32/3.2) | 1 (3.0/3.0) |
| EJ | Bladder | 1.3/0.8 | 2 (40/22) | 4 (1.8/0.5) | 2 (10/4.1) | 5 (36/8.0) | 8 (1.6/0.2) | ND | 1 (30/22) | ND | 5 (22/4.1) | 2 (40/20) |
| SELS | Lung | 18.2/18.6 | 2 (2.2/1.0) | 2 (1.0/0.45) | 2 (12/5.0) | 1 (1.0/1.0) | 1 (0.1/0.1) | 1 (1.5/1.4) | 3 (4.0/1.3) | 1 (0.3/0.3) | 1 (3.0/2.5) | 5 (100/20) |
| MCF-7 | Breast | 11.0/7.3 | 5 (23/4.5) | 2 (6.0/3.0) | 1 (100/100) | 2 (20/10) | 3 (0.9/0.3) | 7 (3.2/0.45) | ND | 1 (0.2/0.2) | 2 (0.4/0.2) | 1 (100/100) |
| MD-MB 231 | Breast | 5.5/8.6 | 9 (16/1.8) | 4 (1.6/0.4) | 1 (30/30) | 2 (12/6.0) | 6 (6.0/1.0) | 4 (0.36/0.09) | ND | 1 (0.3/0.3) | ND | 1 (100/100) |

[a]Sensitivity ratio: $ID_{50}$C–M/$ID_{50}$H–S. $ID_{50}$ is the dose required to inhibit colony formation by 50%. The values were derived from dose-response curves as shown in **Figure 1**, and are given in $\mu g\,ml^{-1}$. [b]$PE_{C-M}/PE_{H-S}$. [c]ND: Not determined. [d]$ID_{50}$ C–M/$ID_{50}$ H–S.

which shows results obtained for tumour A.F. treated with DTIC and CCNU, and for tumour G.A. treated with VBL and BLE. The ratios between the $ID_{50}$ values obtained with the two methods were again calculated. The results (Table II) also show that the biopsy specimens tended to exhibit higher sensitivities when tested by the H–S than by the C–M method. Thus, in 21 out of 35 cases the apparent sensitivity was higher in the H–S method, in 11 cases it was approximately equal, and only in 3 cases was it found to be lower.

### *Effect on growth and chemosensitivity of some factors which differ in the two assays*

In attempts to elucidate which factors are responsible for the differences observed between the two methods, we measured the sensitivities after introducing systematic alterations in one or both methods. The different factors were altered, singly or in combination. In these experiments we chose to use the cell line KN exposed to cis-platinum, since in this case the discrepancy between the two methods was substantial (ratio about 10) and highly reproducible.

*Supplements in the H–S method* The importance of the multiple supplements in the media of the H–S method for growth of different types of tumour cells has not been clearly documented, and their influence on the chemosensitivity is unknown. First, we tested their effect by comparing the growth of the KN cells in the presence and absence of the enrichments. In these experiments the same medium was used in the under- and overlayers, and the effect was tested both with McCoys' medium and CMRL medium.

Figure 3A shows that colony formation was somewhat improved compared to the standard method when McCoy's medium, with or without supplements, was used both in under- and overlayer. Unexpectedly, when CMRL medium was used in both layers (Figure 3B) the highest PEs were obtained when the supplements were omitted in both layers. It should be noted that certain amounts of some supplements (ascorbic acid, asparagine, calcium chloride, L-serine) are also present in the basic media. Therefore, effects of these supplements are difficult to demonstrate. Nevertheless, the results indicate that some of the supplements used in the CMRL medium not only failed to stimulate growth, but in fact inhibited colony formation of KN cells.

Experiments in which we tested individually some of the supplements in the overlayer showed that inclusion of standard preparation of insulin which

**Table II** Relative chemosensitivity obtained with the H–S method and the C–M method in 9 malignant melanoma biopsies exposed to 5 different drugs

| | | *Sensitivity ratio[a] observed with the drugs* | | | | |
|---|---|---|---|---|---|---|
| *Patient* | *PE (%)* | *DTIC* | *CCNU* | *VBL* | *cis-Pt* | *BLE* |
| A.F. | 0.9/0.7[b] | 8 (1000/120)[d] | 10 (12.5/1.25) | 1 (8.0/8.0) | 5 (15/3.0) | 10 (3.0/0.3) |
| A.H. | 0.6/0.3 | 1 (1000/800) | 100 (12.5/0.125) | 100 (8.0/0.08) | ND[c] | ND |
| K.N | 0.3/0.2 | 3 (900/300) | 0.3 (3.0/10) | 1 (2.5/2.5) | ND | ND |
| M.O. | 0.4/0.3 | 1 (1100/900) | 1 (2.0/2.0) | 1 (0.8/0.8) | 0.3 (10/30) | 11 (8.0/0.7) |
| G.A. | 1.2/0.4 | 0.4 (400/1000) | 1 (7.0/6.0) | 6 (0.25/0.04) | 4 (0.3/0.08) | 2 (2.5/1.2) |
| K.L. | 0.7/0.3 | 10 (2500/250) | 100 (8.8/0.08) | ND | ND | ND |
| H.H | 4.8/3.1 | 1 (1200/1200) | 10 (400/40) | 20 (2.0/0.1) | 1 (2.0/2.0) | ND |
| R.M. | 1.0/0.4 | 2 (2000/1100) | 1 (40/40) | 10 (0.2/0.02) | ND | ND |
| F.S. | 1.8/1.7 | 1 (1000/1050) | 7 (28/4.0) | 8 (0.1/0.012) | 4 (4.0/1.0) | 10 (15/1.5) |

[a]Sensitivity ratio: $ID_{50}$C–M/$ID_{50}$H–S. $ID_{50}$ is the dose required to inhibit colony formation by 50%. The values were derived from dose-response curves as shown in **Figure 1**, and are given in $\mu g\,ml^{-1}$.
[b]PE C–M/PE H–S.
[c]ND: Not determined.
[d]$ID_{50}$C–M/$ID_{50}$H–S.

**Figure 3** Influence of medium enrichments in the H–S method on the plating efficiency of KN cells. (●) standard H–S method; (□) McCoy's medium without supplements in both layers; (■) McCoy's medium with supplements in both layers; (△) CMRL medium without supplements in both layers; (▲) CMRL medium with supplements in both layers.

contains preservative (Soehnlen et al., 1980) inhibited the colony forming ability by a factor of approximately 2–3 (Figure 4). Insulin without preservative also inhibited, but to a lesser extent (Figure 4). Omission of ascorbic acid (use of standard formulation as well as ascorbic acid-free CMRL medium) did not influence colony formation. Omission of 2-mercaptoethanol increased colony formation by a factor of ~2.

**Figure 4** Influence of insulin (with and without preservative) in the H–S method on the plating efficiency of KN cells. (●) standard H–S method with insulin with preservative; (×) insulin without preservative; (○) H–S method without insulin.

The effect of the supplements on the sensitivity of KN to cis-platinum was then studied. Omission of supplements from the underlayer (with a standard overlayer) did not alter the chemosensitivity (not shown). In contrast, omission of the standard supplements from the overlayer resulted in a slight decrease in the chemosensitivity (Figure 5). In experiments where insulin, ascorbic acid and 2-mercaptoethanol were omitted separately, no significant change in the sensitivity to cis-platinum was seen (not shown).

*Sera* The H–S method employs horse serum in the overlayer and a mixture of horse serum and heat-inactivated FCS in the underlayer, whereas the C–M method uses FCS that is not heat-inactivated. It was found that the heat-inactivated FCS gave the lowest PE (not shown). The sensitivity of KN cells treated with cis-platinum and cultured in the H–S method under different serum conditions is shown in Figure 6. The sensitivity was highest when the standard H–S method was used, but the difference was small.

*Oxygen concentration and rat erythrocytes (RBC)* A low oxygen concentration (5%) and the presence

**Figure 5** Influence of medium enrichments in the H–S method on the sensitivity of KN cells treated with cis-platinum. (●) standard H–S method; (×) H–S method where supplements in the overlayer were omitted.

**Figure 6** Influence of serum type on the sensitivity of KN cells exposed to cis-platinum and assayed according to the H–S method. (●) standard H–S method; (■) heat-inactivated FCS in both layers; (○) FCS in both layers; (□) horse serum in both layers.

of rat erythrocytes are essential features of the C–M method as they stimulate colony formation in most cases. The influence of these factors on the sensitivity of KN cells to cis-platinum is shown in Figure 7. The sensitivity observed in the C–M method increased when RBC were omitted, whereas

**Figure 7** Influence of oxygen concentration and RBC on the sensitivity of KN cells exposed to cis-platinum and cultivated according to the 2 methods, (a) C–M; (b) H–S. (●) 20% $O_2$, RBC omitted; (○) 5% $O_2$, RBC added; (■) 5% $O_2$, RBC omitted; (□) 20% $O_2$, RBC added.

**Figure 8** Influence of culture vessels on dose-response curves for KN cells treated with cis-platinum. (○) standard C–M method; (□) C–M method employing culture dishes; (●) standard H–S method; (■) H–S method employing culture tubes.

a raise in the oxygen concentration to 20% did not seem to influence the sensitivity. Conversely, when the H–S method was altered in the direction of the C–M method by addition of RBC, a reduced sensitivity was found. Also, in this case the oxygen concentration did not perceptibly influence the observed sensitivity.

*Temperature during solidification. Culture dishes versus tubes* The use of culture tubes (C–M method) rather than Petri dishes (H–S method) involves different procedures during the plating of the cells. While in the H–S method the dishes are usually left for 15–20 min at room temperature, in the C–M method the tubes are immediately put on ice and thereafter into the incubator. This also implies that differences could arise in the pH of the medium, possibly affecting the sensitivity of the cells. To determine the influence of these factors, the procedures were reversed, i.e. in the H–S method the dishes were placed on ice, and in the C–M method the tubes were left at room temperature. These alterations had no effect on the sensitivity of KN cells to cis-platinum.

Also, it was attempted to use dishes in the C–M method and tubes in the H–S method. In the C–M method an underlayer of 0.5% agar in Hams F12 medium without RBC was used. Conversely, in the H–S method the two agar layers were formed in tubes instead of the ordinary dishes. In these cases no replenishing was done. The results in Figure 8 show that the observed sensitivity was independent of whether tubes or dishes were used.

*Plating efficiency as a function of the number of cells seeded*

An essential requirement for a quantitative assay of the colony forming ability of cells is that the number of colonies formed should be proportional to the number of viable cells seeded. This relationship was therefore studied in the two methods.

When the KN cells were plated at low cell densities ($<3\times10^3$ per dish), the colony forming ability in the H–S method was extremely poor (Figure 9). However, above this critical cell density the PE increased markedly, and it was then independent of the number of cells plated. Conversely, the C–M method gave high colony numbers at low cell densities and relatively fewer colonies when more cells were plated (Figure 8), probably due to starvation of the cells under the latter conditions. However, under no conditions did the PEs obtained in the H–S method come close to the PEs in the C–M method. The sensitivity of the KN cells to cis-platinum as a function of the number of cells plated was then studied. Although the different cell densities gave very dissimilar PEs in the growth studies, only minor differences in sensitivity were observed (not shown).

### Discussion

Although soft agar methods have been extensively used during the last years to assess chemosensitivity

**Figure 9** Plating efficiency as a function of number of KN cells plated (in the range 0.5 –10 x $10^3$ cells) in the 2 standard methods. (a) C–M; (b) H–S.

of patients' tumours (Salmon, 1983; von Hoff *et al.*, 1983; Tveit, 1983), a few attempts have been made to examine in detail how culture conditions influence the results. In the present investigation of cell lines and tumour biopsies a comparison was made between the sensitivities to anti-cancer agents measured in two principal soft agar assays (the methods of Hamburger & Salmon and of Courtenay & Mills). Continuous cell lines were employed as they offer several advantages from a methodological point of view (Tveit & Pihl, 1981).

In general, the sensitivity to a series of anti-cancer drugs was found to be higher in the H–S than in the C–M method. These data confirm and extend our previous results on melanoma xenografts exposed to 4 different cytotoxic agents (Tveit *et al.*, 1981*a*). In many cases the chemosensitivity (given by $ID_{50}$ values) differed by a factor of 10 or more. These differences varied both with the cell sample and the particular anti-cancer drug. This is hardly surprising as the observed inhibition of colony formation is the net result of complex interactions involving defective cellular reproduction, impaired synthetic and metabolic functions, loss of repair and recovery capacity, as well as cell death. The factors limiting colony growth may therefore well differ with cell type, and with the mechanism of action of the drug.

Our data show that several factors may influence colony formation in soft agar, but that only a few of these, when tested separately, have any clearly measurable effect on the chemosensitivity results. However, a combination of several factors did seem to influence the sensitivity measurements. Rat erythrocytes did not only considerably increase colony formation not shown, but also appeared to be the most important factor contributing to the observed sensitivity differences.

To our knowledge only Hill and collaborators have performed similar studies as ours. In a study of a colonic carcinoma cell line, exposed to adriamycin or cis-platinum, they found no significant sensitivity differences between the C–M method and the H–S method (Hill, 1983; Hill & Whelan, 1983). It should be noted, however, that these authors actually used a H–S method which had been modified in the direction of the C–M method, both with respect to basic media (Hams F12), sera (FCS), and other supplements that were added (RBC) or omitted (tryptic soy broth, DEAE-dextrane).

The present investigation, as well as our previous studies (Tveit *et al.*, 1981*a*, *b*; Tveit, 1983), indicate that rat erythrocytes stimulate colony formation from human tumour cells to a considerable extent. Furthermore, the present data provide evidence that RBC have a major impact also on the observed chemosensitivity *in vitro*. Several mechanisms may be envisaged to be involved in the reduction of the apparent sensitivity by erythrocytes. In the first place, they have a growth stimulating effect, permitting growth to occur also at low cell densities, thus maintaining linearity between the number of cells plated and the number of colonies formed. When $<10^4$ cells are plated, linearity is not always obtained without erythrocytes present (Tveit *et al.*, 1981*a*). In the absence of erythrocytes the lack of linearity may simulate cell kill and thus give an artificially high sensitivity. Secondly, the possibility exists that erythrocytes may partly neutralize the toxic effects of certain drugs or components liberated by dead cells. Furthermore, they may possibly promote recovery of cells partially damaged by the drug treatment.

Although a low oxygen concentration stimulates colony formation (Courtenay & Mills, 1978; Tveit *et al.*, 1981*a*,*b*; Gupta & Krishan, 1982; Joyce & Vincent, 1983; Sridhar *et al.*, 1983), we were unable to demonstrate any clear effect of the oxygen concentration on the chemosensitivity, in contrast to the findings of Gupta & Krishan (1982).

Systematic studies of the role of the supplements used in the H–S method showed that they influenced the sensitivity to cis-platinum only to a minor extent. However, the studies demonstrated that the use of the enrichments actually inhibited cell growth, and they thus seem to be unnecessary and useless. In our opinion they should be omitted unless it can be definitely shown that they are of value in the particular tumour type studied.

Other factors may also contribute to the observed differences in sensitivity between the H–S method and the C–M method. The type of serum used may be one factor (Figure 6) and the composition of basic media (CMRL, McCoy's, Hams F 12) may well influence sensitivity to certain anti-cancer agents.

A possibility that must be considered is that a selection of certain tumour cell subpopulations may occur during cultivation of tumour cells. It is not inconceivable that different subpopulations may have different sensitivities to certain drugs. We are presently investigating this possibility.

The effects of culture conditions on the apparent chemosensitivity observed in this study are not trivial, and cannot be overlooked as they may have clinical implications. Thus, they imply that chemosensitivity data obtained in different laboratories cannot be directly compared unless they have been carried out under strictly standardized conditions. Moreover, our data show that the measured chemosensitivity does not necessarily reflect the inherent cellular chemosensitivity. Although we have so far demonstrated chemosensitivity differences for a limited number of tumour types only, there is no reason to believe that these represent exceptions. Similar discrepancies will probably be revealed for other forms of tumour. It follows that criteria for sensitivity and resistance should be established for each method. Previous failure to realize this may account for some of the disagreements observed between *in vitro* and clinical chemosensitivity data.

The skilful technical assistance of Mrs. Hanne Kleppe Høifødt is gratefully acknowledged. This work was supported by the Norwegian Cancer Society and the Norwegian Society for Fighting Cancer.

**References**

COURTENAY, V.D. & MILLS, J. (1978). An *in vitro* colony assay for human tumours grown in immune-suppressed mice and treated *in vivo* with cytotoxic agents. *Br. J. Cancer*, **37**, 261.

DENDY, P.P. & HILL, B.T. (1983). *Human Tumour Drug Sensitivity Testing* In Vitro. *Techniques and Clinical Applications*. Academic Press, London.

GUPTA, V. & KRISHAN, A. (1982). Effect of oxygen concentration on the growth and drug sensitivity of human melanoma cells in soft-agar clonogenic assay. *Cancer Res.*, **42**, 1005.

HAMBURGER, A.W. & SALMON, S.E. (1977). Primary bioassay of human tumor stem cells. *Science*, **197**, 461.

HASTINGS, R.J. & FRANKS, L.M. (1983). Cellular heterogeneity in a tissue culture cell line derived from a human bladder carcinoma. *Br. J. Cancer*, **47**, 233.

HILL, B.T. (1983). An assessment of *in vitro* drug sensitivity tests comparing non-clonogenic method with various clonogenic assay procedures, using continuous tumour cell lines. *Proc. 13th Int. Cong. Chemother.*, **224**, 67.

HILL, B.T. & WHELAN, R.D.H. (1983). Attempts to optimize colony-forming efficiencies using three different survival assays and a range of human tumour continuous cell lines. *Cell Biol. Int. Rep.*, **7**, 617.

JOYCE, R.M. & VINCENT, P.C. (1983). Advantage of reduced oxygen tension in growth of human melanomas in semi-solid cultures: Quantitative analysis. *Br. J. Cancer*, **48**, 385.

McKINTOSH, F.R., EVANS, T.L. & SIKIC, B.I. (1981). Methodologic problems in clonogenic assays of spontaneous tumors. *Cancer Chemother. Pharmacol.*, **6**, 205.

SALMON, S.E. (1983). Clinical correlations of *in vitro* drug sensitivity using the clonogenic assay. In *Human Tumour Drug Sensitivity Testing* In Vitro. *Techniques and Clinical Applications*. p. 291. (Ed. Dendy & Hill). Academic Press, London.

SALMON, S.E. (1984). Human tumor colony assay and chemosensitivity testing. *Cancer Treat. Rep.*, **68**, 117.

SELBY, P., BUICK, R.N. & TANNOCK, I. (1983). A critical appraisal of the "human tumor stem-cell assay". *N. Engl. J. Med.*, **308**, 129.

SOEHNLEN, B., YOUNG, L., LIU, R. (1980). Standard laboratory procedures for *in vitro* assay of human tumor stem cells. In *Cloning of Human Tumor Stem Cells*. p. 331 (ed Salmon). Alan R. Liss Inc., New York.

SRIDHAR, K.S., PLASSE, T.F., HOLLAND, J.F., SHAPIRO, M. & OHNUMA, T. (1983). Effects of physiological oxygen concentration on human tumor colony growth in soft agar. *Cancer Res.*, **43**, 4629.

TVEIT, K.M. (1983). Evaluation of the Courtenay assay for drug sensitivity prediction *in vivo*. In *Human Tumour Drug Sensitivity Testing* In Vitro. *Techniques and Clinical Applications*. p. 291. (Ed. Dendy & Hill). Academic Press, London.

TVEIT, K.M., FODSTAD, Ø., JOHANNESSEN, J.V. & OLSNES, S. (1980*a*). A human melanoma cell line established from xenograft in athymic mice. *Br. J. Cancer*, **41**, 724.

TVEIT, K.M., FODSTAD, Ø., OLSNES, S. & PIHL, A. (1980*b*). *In vitro* sensitivity of human melanoma xenografts to cytotoxic drugs. Correlation with *in vivo* chemosensitivity. *Int. J. Cancer*, **26**, 717.

TVEIT, K.M., ENDRESEN, L., RUGSTAD, H.E., FODSTAD, Ø. & PIHL, A. (1981*a*). Comparison of two soft-agar methods for assaying chemosensitivity of human tumours *in vitro*: Malignant melanomas. *Br. J. Cancer*, **44**, 539.

TVEIT, K.M., FODSTAD, Ø. & PIHL, A. (1981*b*). Cultivation of human melanomas in soft agar. Factors influencing plating efficiency and chemosensitivity. *Int. J. Cancer*, **28**, 329.

TVEIT, K.M., FODSTAD, Ø, LOTSBERG, J., VAAGE, S. & PIHL, A. (1982). Colony growth and chemosensitivity *in vitro* of human melanoma biopsies. Relationship to clinical parameters. *Int. J. Cancer*, **29**, 533.

TVEIT, K.M. & PIHL, A. (1981). Do cell lines *in vitro* reflect the properties of the tumours of origin? A study of lines derived from human melanoma xenografts. *Br. J. Cancer*, **44**, 775.

VON HOFF, D.D., CLARK, G.M., STOGDILL, B.J. & 7 others. (1983). Prospective clinical trial of a human tumor cloning system. *Cancer Res.*, **43**, 1926.